# Highly Stable Tetra-Phenolato Titanium(IV) Agent Formulated into Nanoparticles Demonstrates Anti-Tumoral Activity and Selectivity

**DOI:** 10.3390/molecules201018526

**Published:** 2015-10-09

**Authors:** Sigalit Meker, Ori Braitbard, Katrin Margulis-Goshen, Shlomo Magdassi, Jacob Hochman, Edit Y. Tshuva

**Affiliations:** 1 The Institute of Chemistry, The Hebrew University of Jerusalem, Jerusalem 91904, Israel; E-Mails: sigi.meker@gmail.com (S.M.); katymargulis@gmail.com (K.M.-G.); magdassi@mail.huji.ac.il (S.M.); 2 Department of Cell and Developmental Biology, Alexander Silberman Institute of Life Science, The Hebrew University of Jerusalem, Jerusalem 91904, Israel; E-Mails: ori.braitbard@mail.huji.ac.il (O.B.); hochman@mail.huji.ac.il (J.H.)

**Keywords:** titanium(IV), phenolato ligands, cisplatin, cytotoxicity, anti-tumor, cisplatin resistant cells

## Abstract

Titanium(IV) complexes exhibit high potential as anti-tumor agents, particularly due to their low intrinsic toxicity and cytotoxicity toward cisplatin resistant cells. Nevertheless, Ti(IV) complexes generally undergo rapid hydrolysis that previously hampered their utilization as anticancer drugs. We recently overcame this difficulty by developing a highly stable Ti(IV) complex that is based on tetra-phenolato, hexadentate ligand, formulated into organic nanoparticles. Herein we investigated the activity of this complex *in vitro* and *in vivo*. Although inactive when tested directly due to poor solubility, when formulated, this complex displayed (a) high cytotoxicity toward cisplatin resistant human ovarian cells, A2780-cp, with resistance factor of 1.1; (b) additive behavior in combination with cisplatin toward ovarian and colon cancer cells; (c) selectivity toward cancer cells as implied by its mild activity toward non-cancerous, fibroblast lung cells, MRC-5; (d) high stability and durability as manifested by the ability to maintain cytotoxicity, even following one week of incubation in 100% aquatic medium solution; and (e) *in vivo* efficacy toward solid tumors of human colon cancer cells, HT-29, in nude mice without any clinical signs of toxicity. These features support the formulated phenolato Ti(IV) complex being an effective and selective anti-tumoral agent.

## 1. Introduction

**Scheme 1:**
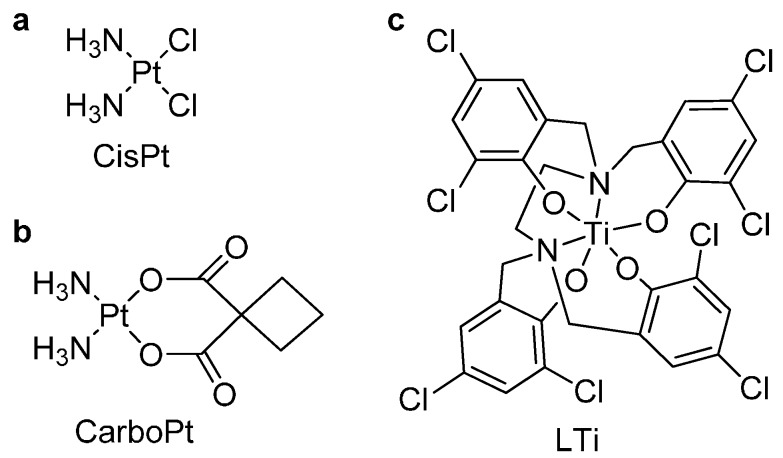
(**a**) Cisplatin; (**b**) Carboplatin; and (**c**) LTi, a cytotoxic Ti(IV) complex that bears tetra-phenolato ligand.

Inorganic titanium(IV) complexes exhibit high potential as non-toxic, effective anti-tumor agents [1,2,3,4,5,6,7,8,9,10,11,12]. They have been investigated as such for several decades due to their high activity toward various cancer cells and low intrinsic toxicity of the titanium metal [2,3,4,5,7,8,13,14], which represent significant advantages over the clinically used cisplatin [1,11,15]. The latter, as well as its derivatives such as carboplatin, suffer from not only severe side effects but also from inherent or acquired resistance of some cell types (Scheme 1) [16,17,18].

The specific Ti(IV) complexes that entered phase I and II clinical trials are budotitane ((bzac)_2_Ti(OEt)_2_) and titanocene dichloride (Cp_2_TiCl_2_); these complexes had demonstrated high activity toward cisplatin resistant cancer cells and low toxicity in pre-clinical trials [3,4,13,19,20,21]. Nevertheless, the complexes failed the trials mainly due to the rapid hydrolysis of the complexes to form inactive, unidentified, polynuclear species [19,20,21,22,23]. Since then, the rapid hydrolysis of Ti(IV) complexes has been an obstacle in utilizing them as potential anti-tumor drugs. Nevertheless, we introduced a third family of cytotoxic Ti(IV) agents that is based on phenolato ligands [24,25,26,27,28,29,30]. Complexes of this type demonstrate high *in vitro* activity toward various cancer cell-lines, *in vivo* efficacy, and markedly improved hydrolytic stability compared with budotitane and titanocene dichloride [31,32]. Furthermore, this family of complexes exhibited strong correlation between their properties and ligand structure [27,28,33,34,35,36]; in particular, phenolato groups, compared with aliphatic ligand derivatives, enhanced the complex cytotoxicity as well as its hydrolytic stability [26]. Under this notion, a Ti(IV) complex LTi that bears tetra-phenolato ligand was synthesized and characterized (Scheme 1) [37,38]. This complex was stable for weeks in 10% D_2_O solutions, but had low solubility and biological accessibility, which impaired its cytotoxicity [38]. Specifically, this complex demonstrated activity toward some murine cell-lines but was inactive toward the more resistant human colon cancer cells, HT-29 [37]. As accessibility is a crucial factor in determining the compound true reactivity [39,40,41], the complex was formulated into organic nanoparticles, by a rapid solvent removal process from volatile oil-in-water microemulsions, which incorporated the complexes in their nanodroplets [37,38,42,43]. As nanoparticles, this complex also demonstrated high cytotoxicity toward HT-29 cells, complete solubility in water and high hydrolytic stability, thus overcoming the difficulties encountered with the previously known Ti(IV) complexes.

Herein, we further examine the potential of this promising compound in its dispersible form for anticancer therapy. Profound *in vitro* studies toward cancerous and non-cancerous cells, as well as those toward cisplatin-sensitive and -resistant cells—alone and in combination with platinum drugs, and evaluation following pre-incubation in medium—all point to high stability, selectivity, and general efficacy of the reported compound. Additionally, *in vivo* measurements further support that the compound has high potential as an anticancer drug.

## 2. Results and Discussion

LH_4_ and the complex LTi were synthesized according to published procedures [38,44]. LTi was then formulated into nanoparticles of unified size via rapid solvent evaporation from microemulsions, as previously described [38].

### 2.1. Cytotoxicity toward Cisplatin Resistant Cells

**Figure 1:**
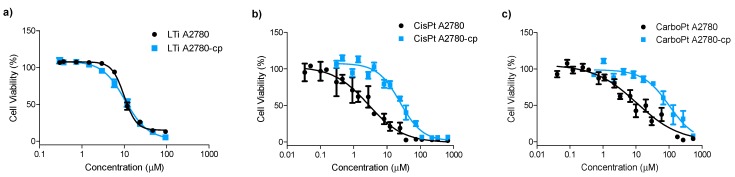
Dependence of cell viability on administered concentration of: (**a**) LTi, (**b**) cisplatin, and (**c**) carboplatin toward human ovarian sensitive A2780, and resistant to cisplatin A2780-cp cancer cell lines.

Resistance to cisplatin leads in some cases to cross-resistance to additional drugs or treatments, therefore overriding cisplatin resistance is extremely valuable for the clinical utility of newly developed drugs. Thus, the cytotoxicity of LTi was measured toward human ovarian cisplatin-sensitive, A2780, and its equivalent cisplatin-resistant, A2780-cp, cancer cell lines. A2780-cp cells, in addition to cisplatin, have cross resistance toward alkylation agent Melphalan, intercalating DNA agent Doxorubicin, and irradiation, due to an increased ability to repair DNA damage as well as cytogenetic abnormalities that include altered DNA damaging recognition and inefficiency of the mismatch repair (MMR) pathway [45,46,47]. The activity of the LTi compared with cisplatin and carboplatin was measured via the methylthiazolyldiphenyl-tetrazolium bromide (MTT) assay, as previously described (Figure 1, Table 1) [48].

LTi demonstrated high anti-proliferative activity toward both the sensitive and the resistant cell-lines, with resistance factor of 1.1. The small resistance factor, *i.e.*, similar activity toward both sensitive and resistant cells, implies the ability of LTi to override cisplatin resistance. Although cisplatin demonstrated slightly higher activity than LTi toward the sensitive line A2780, its activity decreased 11-folds toward the resistant line, A2780-cp [47]. The clinically approved carboplatin was less active than LTi toward both cell lines and demonstrated a resistance factor of 10 [47], similarly to the parent Pt drug, thus supporting the notion that both operate by related mechanisms [49]. These results overall imply differences in the mechanism of action of the titanium complex compared with that of the platinum compounds, where the Ti(IV) complex is capable of overriding the abnormalities related to the DNA repair pathways in the A2780-cp cells [47]. Nevertheless, it cannot be determined unequivocally at this point whether DNA is indeed the target for LTi as it is for the platinum drugs.

**Table 1:** Relative IC_50_ (μM) values toward human ovarian cisplatin-sensitive A2780, and -resistant A2780-cp cancer cell lines and resistance factors.

Complex	IC_50_ (μM)		Resistance Factor ^a^
	A2780	A2780-cp	
LTi	9 ± 1	10 ± 4	1.1
Cisplatin	3 ± 2	33 ± 2	11
Carboplatin	21 ± 14	210 ± 90	10

^a^ Resistance factor is the ratio between IC_50_ value toward the resistant cell-line, A2780-cp, and IC_50_ value toward the sensitive cell line, A2780. IC_50_ values presented are the mean ± SD of at least three independent experiments conducted on different days, each with at least three replicates.

### 2.2. In Vitro Combination with Platinum Drugs

**Figure 2:**
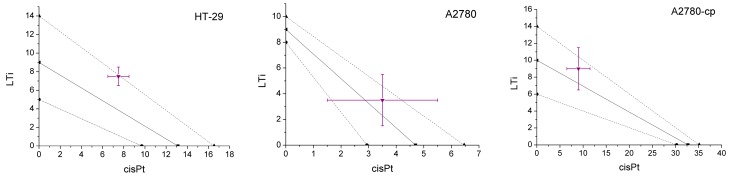
Isobolographic analysis of the cytotoxicity of the combination of LTi and cisplatin, at 1:1 ratio, toward human colon HT-29, ovarian cisplatin-sensitive A2780, and -resistant A2780-cp cell lines. The IC_50_ values of the compounds alone are the axial points, connected to provide the additive line with its error range. Results within the additive range indicate additive behavior, those below indicate synergism, and those above indicate antagonism.

To further examine the differences in the mechanisms of action of LTi and the platinum compounds, their activity as a combination was measured toward HT-29, A2780, and A2780-cp cell-lines. The compounds were administered to the cells as a DMSO/medium solution at a 1:1 ratio. The combination was incubated with the cells for three days and cell viability was measured by the MTT assay. The IC_50_ of the combination was compared with those of each compound when administered alone, and the isobolographic plots are presented in Figure 2 [50,51]. Isobolographic analysis is a method to identify the nature of the interactions of two potential drugs, whether they operate synergistically (benefit one another), additively (work independently), or antagonistically (interfere with one another). The IC_50_ values of the drugs when administrated alone are plotted as axial points of the axes, connected by the additive isobole and its accompanying error range. The results of the experiments are presented as the concentration of both compounds at the calculated IC_50_ point of the combination.

The combination of LTi and cisplatin, at a ratio of 1:1, demonstrated mostly additive behavior toward all lines examined. These results are in good agreement with the notion of different mechanisms of action for cisplatin and the titanium complex. Nevertheless, the activity of 1:1 combination of carboplatin and LTi demonstrated antagonistic behavior toward both of the ovarian, A2780 and A2780-cp cell-lines, with borderline additive effect toward HT-29 cells (Supplementary Figure S1). This does not necessarily result from related mechanisms of the two drugs, but may reflect the effect of indirect parameters such as solubility and cellular penetration. Interestingly, in a previous study on combination of diamino bis-phenolato Ti(IV) complexes and cisplatin [52], one Ti(IV) complex had exhibited highly synergistic effect with cisplatin toward HT-29 cells, whereas a different Ti(IV) derivative showed a clear antagonistic effect. These results overall suggest that the particular derivative selected for treatment is of importance, as ligand substitution may affect cellular penetration, solubility, and interactions with a combined drug. Nevertheless, the results altogether support the utility potential of LTi, both alone and in combinations, particularly with cisplatin, the more effective of the two platinum drugs tested.

### 2.3. Activity Following Incubation in Cell Growth Media

**Figure 3:**
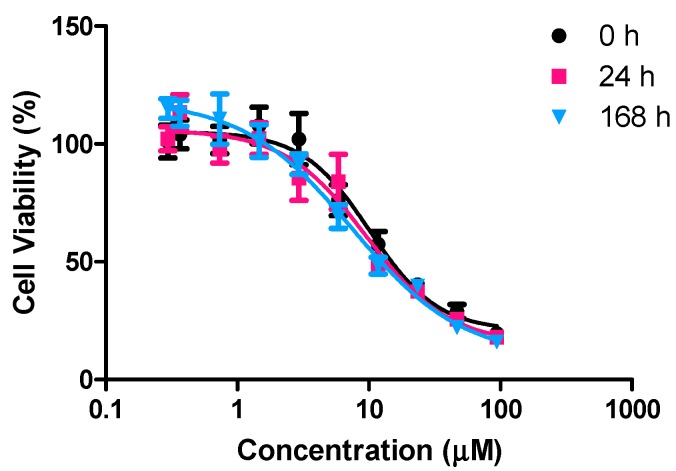
Dependence of human colon HT-29 cell viability on administered concentration of LTi following pre-incubation in complete cell growth medium for varying periods (h) prior to administration to the cells. IC_50_ values (μM), as the mean ± SD of at least three independent experiments conducted on different days, each with at least three replicates: 0 h: 9 ± 4; 24 h: 9 ± 4; 168 h: 8 ± 2.

The Ti(IV) complex LTi has previously shown significantly higher hydrolytic stability compared with other phenolato Ti(IV) complexes. This complex demonstrated resistance to hydrolysis for weeks in comparative measurements that included exposure to 10% D_2_O solutions [38]. To evaluate its hydrolytic stability in conditions that more closely mimic the biological environment, the complex as nanoparticles was exposed to complete RPMI-1640 cell growth media, including 10% fetal bovine serum and 1% antibiotics, for varying periods up to one week (168 h), at 37 °C, and 5% CO_2_. Consequently, the complex was incubated for additional three days with HT-29 human colon cells and its effect on cell viability was measured by the crystal violet assay [53] (Figure 3).

LTi demonstrated high anti-proliferative activity toward HT-29 cells, maintaining its activity even following one week of exposure to complete medium, which substantially exceeds the standard period of incubation with cells in *in vitro* studies. This result is noteworthy considering that previously known titanium compounds demonstrated substantial activity decrease following 5–24 h in medium [31,36]. This observation is consistent with the observed high hydrolytic stability of the complex in aquatic solutions. This implies a long shelf life and durability of this complex.

### 2.4. Selectivity to Cancer Cells

**Figure 4:**
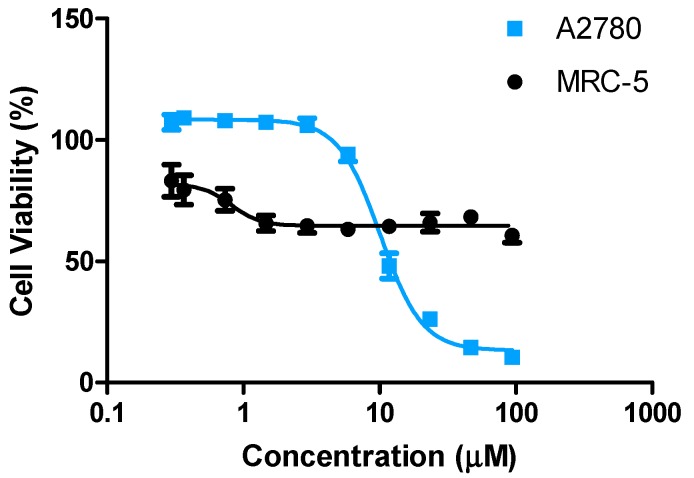
Anti-proliferative activity of LTi toward non-cancerous human lung MRC-5 cells, and human ovarian cancer A2780 cells demonstrated for comparison. IC_50_ values could not be calculated because the compound did not reach 50% inhibition of cell viability.

The selectivity of the anti-proliferating activity of LTi was investigated by measuring its effect on cell viability of MRC-5, non-cancerous, human lung fibroblast cells (Figure 4) [54]. LTi affected moderately the MRC-5 cell viability, thus implying that the anti-proliferative activity of LTi can be selective toward cancer cells.

### 2.5. In Vivo Anti-Tumor Activity

**Figure 5:**
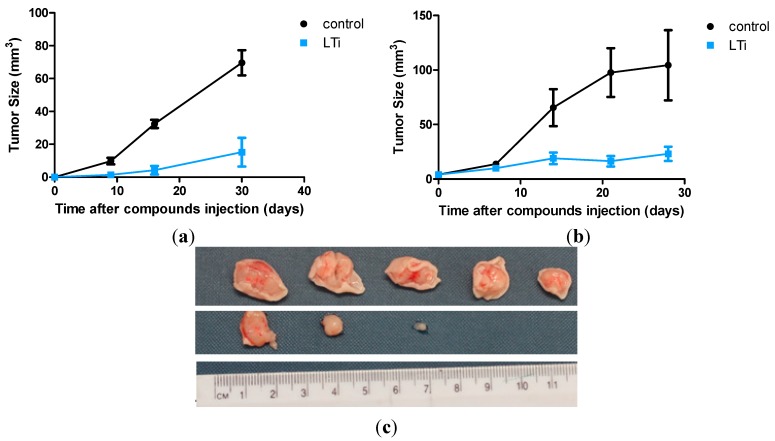
LTi reduces the development of solid tumors from colon cancer cells: immune deficient (nude) mice (**a**) N = 5; (**b**) N = 10 were inoculated S.C. with 5 × 10^6^ HT-29, human colon adenocarcinoma cells and subjected to IP injection of LTi (100 µg/0.2 mL), every other day for three weeks. Control mice were injected with PBS. (**c**) Solid tumors at the termination of the experiment. Upper panel: control mice; Lower panel: treated mice (two-fifths were devoid of tumors).

*In vivo* experiments were conducted to demonstrate the anti-tumorigenic effect of LTi. First, a group of five mice inoculated with human colon adenocarcinoma cells and injected with LTi demonstrated significant decrease in tumor growth relative to control untreated mice (Figure 5). At the end of the experiment, the tumors were taken out for evaluation; whereas the average weight of the tumors developed in the control group was 0.57 ± 0.16 g, that of the treated mice was 0.13 ± 0.18 g, where two of the five treated mice did not develop any tumor. These results were repetitive in a second corroboration experiment, which was conducted with a larger group of 10 mice, out of which three did not develop tumors (Figure 5). These findings clearly demonstrate an anti-tumorigenic effect of LTi (Figure 5). Significantly, no clinical signs of toxicity were observed in the mice treated with LTi, thus emphasizing the potential efficiency and safety of this anti-tumoral agent.

## 3. Experimental Section

### 3.1. Ligand and Complex Synthesis

Ligand LH_4_ and the complex LTi were synthesized according to published procedures [38,44]. LTi was formulated into nanoparticles by evaporation of microemulsions to form nanopowder of LTi, as previously described [38,42].

### 3.2. Materials

Cisplatin crystalline, carboplatin, methylthiazolyldiphenyl-tetrazolium bromide ≥97.5% suitable for cell culture, and crystal violet 1% aqueous solution, were purchased from Sigma-Aldrich Chemical Company Inc (Sigma-Aldrich, St. Louis, MO, USA).

### 3.3. In Vitro Cytotoxicity Assays

Cytotoxicity was measured on HT-29, human colon cells obtained from ATCC Inc (Manassas, VA, USA); A2780, A2780-cis, cisplatin-sensitive and -resistant human ovarian cells; and MRC-5 non-cancerous human fibroblast lung cells, all obtained from ECACC Inc (Salisbury, UK). Cytotoxicity was measured using the methylthiazolyldiphenyl-tetrazolium bromide (MTT) assay [48] or by crystal violet assay [53]. Cells (0.6 × 10^6^) in medium containing 1% penicillin/streptomycin antibiotics; 1% l-glutamine; 10% fetal bovine serum (FBS); and 88% medium RPMI-1640, all purchased from Biological Industries Inc., were seeded into a 96-well plate and allowed to attach for a day. The cells were consequently treated with the reagent tested at 10 different concentrations. Solutions of cisplatin and carboplatin were prepared by dissolving 1–8 mg in 100–200 μL of DMSO, with further diluting to obtain the 10 different concentrations. Organic solutions were then further diluted with medium to give 10% DMSO solutions, followed by applying 10 μL of each final solution to wells already containing 200 μL of the above solution of cells in the medium; giving final concentrations of the complexes up to 1000 mg/L, in 0.5% DMSO solutions. LTi was diluted similarly into RPMI-1640 medium without any organic solvent. Combination measurements were conducted similarly by preparing stock solutions of LTi and the platinum compound in 1:1 ratio and diluted it to 10 different concentrations. Control wells were treated with similar amounts of DMSO or RPMI-1640.

MTT assay: After a standard of 3 days incubation at 37 °C in 5% CO_2_ atmosphere, MTT (0.1 mg in 20 μL) was added to the cells; cells were then incubated for additional 3 h with MTT. The MTT solution was removed, and the cells were dissolved in 200 μL isopropanol. The absorbance at 550 nm was measured by a Bio-Tek EL-800 microplate reader spectrophotometer.

Crystal violet assay: After a standard of 3 days incubation at 37 °C in 5% CO_2_ atmosphere, the cells medium was removed and the cells were fixated by incubating them in 200 μL formaldehyde 37%, at room temperature, for 5 min. The formaldehyde was removed and 100 μL of 1% crystal violet aqueous solution was added. Following 20 min of incubation of the cells with crystal violet at room temperature, the crystal violet solution was removed and the cells were washed twice with water. The cells were dissolved in 200 μL of methanol and the absorbance at 570 nm was measured by a Bio-Tek EL-800 microplate reader spectrophotometer.

Each measurement was repeated at least 3 × 3 times, namely, three repeats per plate, all repeated at least 3 times on different days (9 repeats altogether). Relative IC_50_ values were determined by a non-linear regression of a variable slope (four parameters) model. IC_50_ values presented in text as the mean ± SD of at least three independent experiments.

### 3.4. Activity Following Incubation in Cell Growth Media

LTi activity following pre-incubation in complete cell growth media was accomplished by diluting LTi dispersions in cell growth media containing 1% penicillin/streptomycin antibiotics, 1% l-glutamine, 10% fetal bovine serum (FBS), and 88% medium RPMI-1640, as described above. The dispersions were incubated at 37 °C in 5% CO_2_ atmosphere for 0, 24, and 168 h before applying them to cells that were seeded as detailed above. Cells were incubated with the dispersions for 3 additional days and cell viability was measured by crystal violet assay, as described.

### 3.5. In Vivo Anti-Tumor Activity

*In vivo* analysis was conducted as followed: Nude Balb/C mice (6–8 weeks old females) were obtained from Harlan (Israel), held in a specific-pathogen-free (SPF) facility (AAALAC accreditation #1285) and treated in accordance with the national institutes of health (NIH) guidelines and approval by the institutional committee for ethics in animal experimentation. Mice were injected S.C. with 5 × 10^6^ HT-29, human colon adenocarcinoma cells. Starting 24 h after the challenge, mice were subjected to IP injections of LTi, every other day for 3 weeks. Mice were followed up to 28 days post inoculation.

## 4. Conclusions

LTi formulated into nanoparticles is a remarkably effective anti-tumoral agent. Not only is it active *in vitro* toward a number of cells, but it is also active toward cisplatin-resistant cells, and shows *in vivo* efficacy. The high efficacy is accompanied by low toxicity as expected of the titanium metal, as manifested by the inactivity toward non-cancerous cells as well as the absence of clinical signs of toxicity in the treated animals. Moreover, the superiority of LTi particularly lays in the high hydrolytic stability of the compound, especially as no change in activity was obtained after up to a week in complete aquatic medium, implying a long shelf life of this complex, which is essential for therapeutic applications. The possibility of additive effects when combined with known drugs adds to the high potential of this complex for use in a clinical setting.

It is interesting to recall LTi was inactive when administered directly as is, due to insufficient solubility; thus, the nano-formulation enabled the compound to demonstrate its true behavior [38]. It is thus obvious that solubility and general biological accessibility are crucial factors in the development of any potential drugs, where nano-formulations presents an effective way to overcome such limitations.

Our preliminary studies suggest a different mechanistic pathway for LTi than that of cisplatin, as may be deduced by the high activity of LTi on cisplatin-resistant cells and by the additive influence of the drugs in *in vitro* combination analyses that generally indicate independent operations. Nevertheless, full mechanistic determination is still lacking. Thus, due to the incredibly high potential of LTi as displayed by the results presented herein, intensive profound mechanistic analyses as well as development of related derivatives are underway in our laboratory.

## Supplementary Materials

Supplementary Materials for this paper include isobolographic plots of the combination of LTi and carboplatin. Supplementary materials can be accessed at: http://www.mdpi.com/1420-3049/20/10/18526/s1.
