# Supplementary material for: Highly Stable Tetra-Phenolato Titanium(IV) Agent Formulated into Nanoparticles Demonstrates Anti-Tumoral Activity and Selectivity
Source: Molecules. 2015 Oct 9;20(10):18526–38. doi: 10.3390/molecules201018526 (PMC6331959; doi:10.3390/molecules201018526)
Supplement: Supplementary file 1 [file molecules-20-18526-s001.pdf]

Supplementary Material

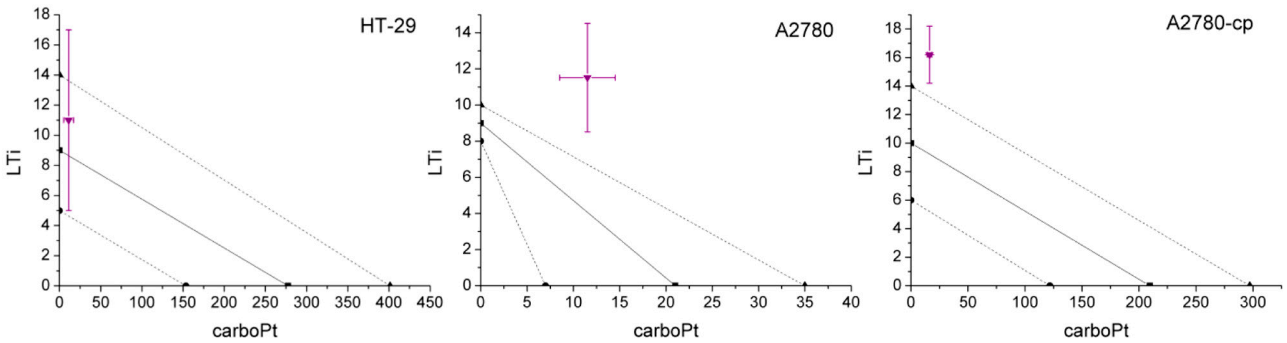

Figure S1. Isobolographic analysis of the cytotoxicity of the combination of LTi and carboplatin, at 1:1 ratio, toward human colon HT-29, ovarian cisplatin-sensitive A2780, and -resistant A2780-cp cell lines. The IC_{50} values the compounds alone are the axial points, connected to provide the additive line with its error range. Results within the additive range indicate additive behavior, those below indicate synergism, and those above indicate antagonism.
